# Inference of pan-cancer related genes by orthologs matching based on enhanced LSTM model

**DOI:** 10.3389/fmicb.2022.963704

**Published:** 2022-10-04

**Authors:** Chao Wang, Houwang Zhang, Haishu Ma, Yawen Wang, Ke Cai, Tingrui Guo, Yuanhang Yang, Zhen Li, Yuan Zhu

**Affiliations:** ^1^Department of Surgery, Hepatic Surgery Center, Institute of Hepato-Pancreato-Biliary Surgery, Tongji Hospital, Tongji Medical College, Huazhong University of Science and Technology, Wuhan, China; ^2^Department of Electrical Engineering, City University of Hong Kong, Kowloon, Hong Kong SAR, China; ^3^School of Automation, China University of Geosciences, Wuhan, China; ^4^Hubei Key Laboratory of Advanced Control and Intelligent Automation for Complex Systems, Wuhan, China; ^5^Engineering Research Center of Intelligent Technology for Geo-Exploration, Wuhan, China; ^6^School of Mathematics and Physics, China University of Geosciences, Wuhan, China; ^7^Key Laboratory of Computational Neuroscience and Brain-Inspired Intelligence, Shanghai, China

**Keywords:** microbe-disease, orthologs, essential proteins, deep learning, BiLSTM model

## Abstract

Many disease-related genes have been found to be associated with cancer diagnosis, which is useful for understanding the pathophysiology of cancer, generating targeted drugs, and developing new diagnostic and treatment techniques. With the development of the pan-cancer project and the ongoing expansion of sequencing technology, many scientists are focusing on mining common genes from The Cancer Genome Atlas (TCGA) across various cancer types. In this study, we attempted to infer pan-cancer associated genes by examining the microbial model organism Saccharomyces Cerevisiae (Yeast) by homology matching, which was motivated by the benefits of reverse genetics. First, a background network of protein-protein interactions and a pathogenic gene set involving several cancer types in humans and yeast were created. The homology between the human gene and yeast gene was then discovered by homology matching, and its interaction sub-network was obtained. This was undertaken following the principle that the homologous genes of the common ancestor may have similarities in expression. Then, using bidirectional long short-term memory (BiLSTM) in combination with adaptive integration of heterogeneous information, we further explored the topological characteristics of the yeast protein interaction network and presented a node representation score to evaluate the node ability in graphs. Finally, homologous mapping for human genes matched the important genes identified by ensemble classifiers for yeast, which may be thought of as genes connected to all types of cancer. One way to assess the performance of the BiLSTM model is through experiments on the database. On the other hand, enrichment analysis, survival analysis, and other outcomes can be used to confirm the biological importance of the prediction results. You may access the whole experimental protocols and programs at https://github.com/zhuyuan-cug/AI-BiLSTM/tree/master.

## 1. Introduction

Cancer is a malignant and complex kind of disease that seriously endangers human existence. Because of its rapid spread, early onset, and high death rate, cancer is a disease that is challenging to cure. According to the American Cancer Society, there will be 608,570 cancer-related deaths and 1,898,160 new cases of cancer in the nation in 2021 (Xia et al., 2022). The prevention and treatment of cancer have evolved into a public health issue that requires collective human effort. A growing number of scholars are dedicating themselves to pan-cancer research as it is a hot topic globally. The therapeutic treatment of viral diseases, genetic diseases, and other diseases may be improved by the use of gene therapy (Ma et al., 2020). Therefore, accurate detection of pan-cancer genes is essential for understanding cancer and provides better benefits for its prevention, treatment, and development of anti-cancer drugs, which is relevant from a social and economic perspective (Aromolaran et al., 2021).

Currently, the identification of essential genes is the main source of the issue with pan-cancer associated genes prediction. In previous decades, biological experiments including single gene knockout, conditional knockout, and RNA interference were used as the typical methods for identifying essential proteins. These experimental techniques require lengthy and expensive procedures, and the experimental settings frequently affect the outcomes. The same organism may respond differently to different experimental settings (Zhong et al., 2021). An enormous number of protein-protein interactions (PPI) enriched with gene expression data have been available in recent years benefiting from the advancement of high-throughput technology (Li et al., 2017).

According to the two sides, studies on cancer-related genes can be roughly split into two categories. It is intended to investigate the tissue-specific driver genes, on the one hand. The ideas pertaining to complex network analysis were transferred and utilized to biological network analysis by merging cancer sample data onto biological networks. Each node in the network structure had its level of importance evaluated, and the genes with the highest value were found to be the cancer driver genes. Since genes only selectively express proteins, essential proteins can be used to discover essential genes. Numerous effective network-based techniques have been put forth over years to identify crucial proteins from PIN. The most well-known and straightforward one is degree centrality (DC) (Jeong et al., 2001). According to a molecular theory known as the centrality-lethality rule, the highly linked nodes within the PIN serve as its fundamental structural components and are generally more significant than other nodes (Jeong et al., 2001; Zotenko et al., 2008). Other node topological feature-based methods, such as subgraph centrality (SC) (Estrada and Rodriguez-Velazquez, 2005), eigenvector centrality (EC) (Bonacich, 1987), betweenness centrality (BC) (Joy et al., 2005), closeness centrality (CC) (Wuchty and Stadler, 2003), information centrality (IC) (Stephenson and Zelen, 1989) and others, are also used to identify proteins in addition to DC. These techniques assess each node according to its topological structure. In general, network-based approaches are extensively employed in the early stages since they can predict important proteins directly without the need for further information. However, these techniques feature low recall rates and identification precision due to the abundance of false positive and false negative data in PPI networks (Li et al., 2016). The intrinsic biological importance of necessary proteins is also disregarded by these techniques, which ignores essential proteins with low connectivity (Li et al., 2016). Recent research has attempted to incorporate biological knowledge into network-based techniques, which not only reduce the impact of false positives in PPI data but also significantly increase the prediction accuracy of essential proteins (Li et al., 2012; Zhang et al., 2019; Wang et al., 2021). Ess-NEXG (Wang et al., 2020) and DeepEP (Zeng et al., 2019; Liu et al., 2022b) are two related algorithms for finding essential proteins that have been developed as a result of the rapid growth of deep learning. Other algorithms have also been presented to predict other associations (Zhang et al., 2021; Liu et al., 2022a,b).

On the other side, it seeks to identify potential disease-related genes across a variety of malignancies. Several computational methods have been proposed to uncover pan-cancer related genes or driver module types by integrating multi-omics data across various malignancies (Cao and Zhang, 2016; Zhang and Zhang, 2016, 2017; Yang et al., 2017; Li et al., 2020), which is motivated by the objectives of the cancer genome program named The Cancer Genome Atlas (TCGA) (Weinstein et al., 2013). By combining existing information on cancer from various types of tumors, potential patterns and biological processes are investigated. For example, Park et al. (2016) proposed an algorithm called NTriPath based on matrix decomposition to identify and complement pathogenic gene pathways, which overcomes the limitation of studying a single cancer and can complement the existing set of pathogenic pathway genes in multiple cancers. In order to identify possible pan-cancer related genes, Zhu et al. (2022) combined the network representation method with differential expression analysis.

Geneticists have long noted that functional relationships frequently exist between mutations that result in the same biological manifestation. Utilizing these predictions to connect particular genes to phenotypes opens the door to using similar techniques to directly find new disease genes in the study of human genes. In reverse genetics, it is feasible to infer linked phenotypes based on linkages in functional gene networks (Sommer, 2008). Homologous genes are genes found in several species that descended vertically from a single gene found in the last common ancestor, which is how organisms evolved from a common ancestor. Highly identical DNA sequences between two homologous genes, which may also have the same function, are extremely likely to be found in two animals with very close affinity (Müller, 2003). The concept of homology allows us to more easily study human genes with gene sequences from other species. Similar structures and functionalities are shared by genes that are crucial for life's functions in model organisms. Furthermore, there is mounting evidence that model species are essential for addressing issues connected to the gene variations that underlie human disease. Using model organisms for homology mapping can help us understand human pathogenic genes (Bleackley and MacGillivray, 2011).

Due to its genetic flexibility, small genome size, and manipulability, yeast is one of the model organisms with the highest genetic adaptability. Yeast is a single-cell eukaryote that helps to uncover many fundamental concepts in biology and reveals the activity of human cells. Consequently, yeast is essential for identifying genetic variations in human genes related to illnesses and encoding genetic variations in proteins engaged in multiple pathways. The study revealed a link between the microbiota and associated diseases, and it is crucial to understand the molecular mechanisms of these diseases in order to develop new microbiome-based therapies. Microbiota is the microbial population colonizing multiple organ systems in humans and impacting the outcomes of microbiota-related diseases (Belkaid and Hand, 2014; Sun et al., 2022). Among them, gut microbiota, a dense microbial community in human intestines, has been found closely associated to acute kidney injury (Lei et al., 2022), atherosclerosis (Anto and Blesso, 2022), reduced bone mineral density (Wan et al., 2022), age-related neuroinflammation and cognitive decline (Alsegiani and Shah, 2022), carcinogenesis and cancer immunotherapy resistance (Hersi et al., 2022), and metabolic disorders such as hyperlipidemia, hyperglycemia, hypertension, obesity and diabetes (Beg et al., 2022). Manipulation of the gut microbiota has broad application prospects on diseases. Fecal microbiota transplant (FMT) is one of the microbiome-based therapeutics with clinical application potential in clostridioides difficile colitis, graft-vs.-host disease, and inflammatory bowel disease (Sorbara and Pamer, 2022). In addition, engineered bacteria, postbiotics, and phages are also used as precision microbiome-centered therapies (Bajaj et al., 2022).

Multiple biological data are currently available due to the advancement of sequencing technologies, enabling it to integrate multi-omics data from various tumors to uncover genes associated to pan-cancer. In this study, we use the yeast network to predict human disease genes. We gathered a pathogenic gene set from multiple cancers. Homologous mapping is then utilized to locate the homologs integrating all of the pathogenic genes of ten tumors. We propose a parameter adaptive model for characterizing node representation ability by merging Subcellular localization information, Gene expression data, and Protein Complexes data with the specifically designed topological properties of the PPI network, which is called PSGN score for short. Additionally, the BiLSTM, a LSTM model with adjacency constraint and multiple features, is proposed for the prediction of essential proteins. The yeast genes that are similar to the seed genes are screened as candidate genes using the BiLSTM algorithm. In order to identify the final predicted human pan-cancer associated genes, homolog mapping of these candidate genes was performed.

Comparative experiments were conducted on the publicly accessible PPI data of Yeast, in order to validate the effectiveness of the proposed evaluation PSGN score and the classification results of BiLSTM. We verified the efficacy of the new proposed score by contrasting the performance of PSGN with classic unsupervised approaches including DC, BC, CC, EC NC, LAC, PeC, and WDC. Further, we compared our BiLSTM model to established machine learning techniques like SVM, decision tree, ensemble learning-based methods, and the most recent deep learning-based approach put forth by Zeng et al. (2019). According to the experimental findings, BiLSTM may identify essential proteins with superior overall outcomes than other cutting-edge techniques. Besides, some biological significance experiments were conducted on real datasets, the results validated the effectiveness of the new proposed algorithm from the reverse genetics perspective. The remaining parts are organized as follows. Section 2 presents the material and methods of the new proposed method. Experimental results and discussions are illustrated in Section 3. Finally, Section 4 concludes the work.

## 2. Materials and methods

### 2.1. Datasets

**PPI networks:** among other species, the PPI network dataset of yeast is the most reliable and complete, making it popular for use in evaluating and identifying essential proteins. Therefore, in this study, we also selected the yeast PPI network dataset. The DIP database is used to gather the PPI data of yeast (Xenarios et al., 2002). There are 5,093 proteins and 24,743 interactions in total after subtracting self-interactions and repetitive interactions.

**Essential protein datasets:** A list of essential proteins of yeast were collected from the following databases: MIPS (Mewes et al., 2006), SGD (Cherry et al., 2012), and DEG (Zhang and Lin, 2009). A protein in the yeast protein interaction network is considered as an essential protein if it is marked as essential at least in one database. This data has 1,285 essential proteins, 1,167 of which are included in the PPI network constructed from the DIP database. Hence, we take the 1,167 proteins as essential proteins and the rest 3,926 proteins as non-essential ones.

**Subcellular localization dataset:** the dataset is available in the knowledge channel of COMPARTMENTS database (Binder et al., 2014), which combines the UniProtKB (Magrane, 2011), MGD (Eppig et al., 2012), SGD (Cherry et al., 2012), FlyBase (Mcquilton et al., 2012), and WormBase datasets (Harris et al., 2010). There are 206,831 subcellular localization records in this dataset, which can be further subdivided into 830 categories.

**Protein complex datasets (Luo and Qi**, **2015****):** it is comprised of four real protein complex sets (CM270, CM425, CYC408, and CYC428). Seven hundred and forty-five protein complexes are included in the consolidated dataset.

**Gene Expression Omnibus (GEO) dataset:** GSE3431 derives from GEO and samples 12 time points during each of three yeast successive metabolic cycles (the interval between two time points is 25 min). The dataset contains 36 samples with 6,777 genes.

Online Mendelian Inheritance in Man (OMIM) dataset: we retained only disease-related variants linked to a genetic disorder listed in the OMIM database. Cross-references were used to directly access annotations for each OMIM disease by downloading the DO (Human Disease Ontology) OBO (Open Biological and Biomedical Ontology) file release. Each retrieved leaf DO term connected to a single OMIM was expanded to include all ancestors and the ontological root term. Term expansion was calculated by parsing the OBO file with an impromptu script.

The Cancer Genome Atlas (TCGA) Database: the Human Genome Research Institute (HGRI) and National Cancer Institute (NCI) launched the Cancer Genome Mapping Project in 2006. The database contains more than 20,000 samples from 33 cancer types, including transcriptome expression data, genome variation data, methylation data, clinical data, and others, which can be accessed *via* https://portal.gdc.cancer.gov/exploration.

### 2.2. Overview of the new proposed method

**Figure 1:**
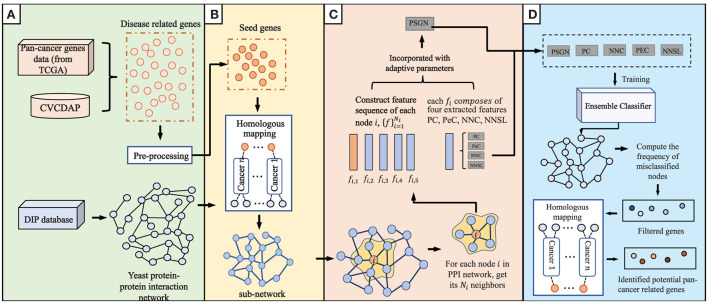
An overview of our proposed method to predict pan-cancer related genes *via* orthologs matching. The main algorithm consists of **(A–D)** four parts. **(A)** data integration and pre-processing. **(B)** Homologous mapping. **(C)** Evaluation of node representation ability in graph. **(D)** Prediction of pan-cancer related genes.

The current proposed method, which consists of four main steps of data integration and pre-processing, homologous mapping, evaluation of node representation ability, and prediction of pan-cancer related genes, is shown in detail in Figure 1.

#### 2.2.1. Data integration and pre-processing

The gene expression data of 10 cancers were obtained from TCGA database, including esophageal carcinoma, pancreatic cancer, lung cancer (lung adenocarcinoma, lung squamous cell carcinoma), breast invasive carcinoma, colon adenocarcinoma, rectum adenocarcinoma, cholangiocarcinoma, gastric cancer and ovarian cancer. Due to the duplications and deletions in the pathogenic genes of each cancer, they are used as experimental data after sorting and deletion. We uploaded the TCGA data of 10 cancers selected in the CVCDAP database (https://omics.bjcancer.org/cvcdap/home.do), successfully generated the pan-cancer related pathogenic gene set, and completed the analysis of the pan-cancer network driving genes with the help of the analysis tool of CVCDAP database. We obtained the data of Yeast protein interaction network on DIP database (https://dip.doe-mbi.ucla.edu/dip/Main.cgi) and downloaded the connection information between Yeast protein nodes directly.

#### 2.2.2. Homologous mapping

We believe that the homologous genes of co-ancestors express themselves similarly. The NCBI Homologene database (https://www.ncbi.nlm.nih.gov/homologene/) compiles homologous gene data for species with complete genome sequencing. In this section, we used Homologene package in the R language, the imported human pathogenic genes were annotated by homology mapping, and the homologous genes of human and yeast genes were taken as seed genes. After identifying the proteins expressed by the seed genes, the interaction network among these yeast proteins can be determined by the STRING database (https://cn.string-db.org/).

#### 2.2.3. Evaluation of node representation ability

Yeast is one of the most genetically model organism. In this study, we firstly explore the essential proteins in the yeast PPI network to further find potential disease related genes. Thus, a new score is defined to evaluate the node representation ability. The PPI network is denoted as graph *G* = (*V,E*), where *V* = {*v*_1_, ⋯ , *v*_*m*_} and *E* = {*e*_*i,j*_, 1 ≤ *i,j* ≤ *m*} represent the node set and edge set of the graph, respectively. Specifically, *v*_*i*_ denotes the *i*-th protein while *e*_*ij*_ denotes protein-protein interaction linkage between protein *v*_*i*_ and *v*_*j*_. |*V*| = *m* represents the number of total proteins within *G*.

The features of our new proposed score considers node-aided biological information, edge-aided biological information and network topological features. We'll go through how to use and integrate this data to create the attributes needed to determine a protein's essentiality in the subsections that follow. The establishment process requires three specific steps.

Step 1: Construction of node represented features

1) Protein complexes score: previous studies indicated that intracellular proteins always tend to connect with their neighbors to form densely connected modules, which are called protein complexes and by this way proteins could take part in more complex and diverse biological activities and functions (Luo and Qi, 2015). Given that essential proteins are crucial in maintaining the main structure and functions in protein complexes (Zotenko et al., 2008), protein complexes data could be used for the identification of essential proteins (Lei et al., 2018).

For the protein *v*_*i*_, the essentiality tends to be higher if it is found in more protein complexes. In order to calculate the protein complexes (PC) score, we do the following:


(1) $$\begin{array}{l} PC\left(i\right)=|Complex\left(i\right)| \end{array}$$

where *Complex*(*i*) denotes the sets of protein complexes including *v*_*i*_, and |*Complex*(*i*)| is the number of protein complexes including *v*_*i*_.

2) Subcellular localization score: it has been proved that proteins must be localized at their appropriate subcellular compartments to perform their desired functions and thus the subcellular localization information is beneficial for the identification of essential proteins (Peng et al., 2015). To ensure the relationships of subcellular localization with the topological features of PPI network, refer to Li et al. (2016), we firstly use the previous feature NNC to sort the proteins within the PPI network, and then calculate the numbers of subcellular location *l* where the top *k%* proteins appear and where the bottom *k%* proteins appear, respectively.

Given the data's false positives, counting proteins at higher rates may result in more errors; as a result, we use *k* = 5 in this work as Li et al. (2016) sets, i.e., that the top/bottom 5% proteins are selected. Besides, we define *T*_*l*_ as the frequency of the localization *l* where the top *k%* proteins appear and *B*_*l*_ as the frequency of the localization *l* where the bottom *k%* proteins appear. Subcellular localization correlation coefficient SLCC(*l*) can be calculated by Equation (2)


(2) $$SLCC(l)=\left\{ \begin{array}{l} 1-\frac{B_{l}}{T_{l}},T_{l}<B_{l};\\ \frac{T_{l}}{B_{l}}-1,otherwise, \end{array}\right.$$

when *T*_*l*_ < *B*_*l*_, it means that more proteins with low NNC values tend to appear in the location *l* and it is assumed that the relationship between the location *l* and the essentiality of proteins is negative. On the other hand, when *T*_*l*_ ≥ *B*_*l*_, there should also be a positive correlation between the location *l* and the essentiality of proteins. When *T*_*l*_ = 0, we set *SLCC*(*l*) as the maximum of $1-\frac{B_{l}}{T_{l}}$ with *T*_*l*_≠0. And when *B*_*l*_ = 0, we set *SLCC*(*l*) as the maximum of $\frac{T_{l}}{B_{l}}-1$ with *B*_*l*_≠0.

Besides, considering that a protein may appear in multiple subcellular locations, take protein *v*_*i*_ for instance, its subcellular localization score *SL*(*i*) could be calculated as the sum of *SLCC*(*l*) of all the subcellular locations where it appears. Moreover, the normalized value *NSL*(*i*) of *SL* for each protein *v*_*i*_ is used by Equation (3)


(3) $$\begin{array}{l} NSL\left(i\right)=\frac{SL\left(i\right)+max\text{\_}SL}{Max\left(SL\left(i\right)+max\text{\_}SL\right)}, \end{array}$$

where *max*_*SL* represents the maximum value of *SL*(*i*) for all the proteins within the PPI network. *Max*in the denominator takes for all the nodes within the PPI network.

In order to strengthen the identification precision of subcellular localization, we combine the NSL score with a network topological feature NNEC that is proposed in Zhu and Wu (2018) and has a good compatibility with biological information. The combined feature is called NNSL for short, for each protein *v*_*i*_, its *NNSL*(*i*) score can be calculated by Equation (4)


(4) $$\begin{array}{l} NNSL\left(i\right)=NSL\left(i\right)\times NNEC\left(i\right), \end{array}$$

where $NNEC\left(i\right)=\underset{j\in \mathbb{N}\left(i\right)}{\sum }NECC\left(i,j\right)$ and *NECC* can be obtained by Equation (5)


(5) $$\begin{array}{l} NECC\left(i,j\right)=\frac{T{\left(i,j\right)}^{3}\times C\left(j\right)}{\prod_{t=\left\{i,j\right\}}\left(d\left(t\right)-1\right)}, \end{array}$$

where *T*(*i, j*) denotes the number of triangles made up of proteins *v*_*i*_ and *v*_*j*_, $C\left(j\right)=\frac{2E_{j}}{d\left(j\right)\left(d\left(j\right)-1\right)}$ is the clustering coefficient of protein *v*_*j*_, *E*_*j*_ is the number of non-repetitive edges consisting of all nearest neighbors of *v*_*j*_. *d*(*t*) denotes the degree for protein *t*, for *t* = *i* or *j*.

Step 2: Construction of edge represented features

Gene expression data is a type of biological information that has been utilized for a long time to compute edge correlations and identify essential proteins. *PeC* is a method that combines gene expression data with edge clustering coefficient *ECC* in order to reduce the impact of false positives on the PPI network. As a result, we apply *PeC* in this study to extract pertinent information from gene expression data. For a protein *v*_*i*_, its PeC score *PeC*(*i*) can be computed by Equation (6)


(6) $$\begin{array}{l} PeC\left(i\right)=\underset{j\in \mathbb{N}\left(i\right)}{\sum }ECC\left(i,j\right)\times PCC\left(i,j\right), \end{array}$$

where *ECC*(*i, j*) is the edge coefficient between edge *e*_*i,j*_, *PCC*(*i, j*) is the Pearson's correlation coefficient of a pair of proteins (*v*_*i*_ and *v*_*j*_). *s* denotes the length of the gene expression data, which can be calculated by Equation (7)


(7) $$\begin{array}{l} PCC\left(i,j\right)=\frac{1}{s-1}\overset{s}{\underset{t=1}{\sum }}\left[\frac{g\left(i,t\right)-ḡ\left(i\right)}{\sigma \left(i\right)}\right]\times \left[\frac{g\left(j,t\right)-ḡ\left(j\right)}{\sigma \left(j\right)}\right], \end{array}$$

where *g*(*i, t*) and *g*(*j, t*) are the expression level of *v*_*i*_ and *v*_*j*_ in the sample time *t* under a specific condition, ḡ(*i*) and ḡ(*j*) represent the mean expression level of *v*_*i*_ and *v*_*j*_, and σ(*i*) and σ(*j*) represent the standard deviation of expression level of *v*_*i*_ and *v*_*j*_, respectively.

To extract the topological information of proteins within the PPI network, it is necessary to construct an effective feature representing the network structures of the nodes and connections with neighbors. Network centrality (NC) is a representative topology based method widely used for predicting essential proteins (Wang et al., 2012). Hence, we choose it for network topological feature construction. For the protein *v*_*i*_, its network centrality *NC*(*i*) can be calculated as the sum of edge clustering coefficients *ECC*(*i, j*) of each edge *e*_*i,j*_ connected with *v*_*i*_ by Equation (8)


(8) $$\begin{array}{l} \begin{array}{c} NC\left(i\right)=\underset{j\in \mathbb{N}\left(i\right)}{\sum }ECC\left(i,j\right)\\ =\underset{j\in \mathbb{N}\left(i\right)}{\sum }\frac{T\left(i,j\right)}{min\left(d_{i}-1,d_{j}-1\right)}, \end{array} \end{array}$$

where ℕ(*i*) is the set of nodes which directly connect with protein *v*_*i*_.

In order to match other features based on biological information, here we use the normalized NC value (denoted as NNC) for each protein. Then for *v*_*i*_, its normalized value *NNC*(*i*) is defined by Equation (9)


(9) $$\begin{array}{l} NNC\left(i\right)=\frac{NC\left(i\right)}{max\left(NC\left(i\right)\right)}, \end{array}$$

where *max*(*NC*(*i*)) denotes the maximum NC value of all the proteins in the graph *G*, and the value of *NNC*(*i*) will be normalized between 0 and 1.

Step3 : Feature integration by liniear model with adaptive parameters

The structure of hetergeous feature integration involves Protein complex *PC*(*i*), Subcellular localization *NNSL*(*i*), Gene expression *PeC*(*i*) and Network topology *NNC*(*i*) multiple information (PSGN). Here, we reconcile these features using a linear model in order to fully integrate this information. Take protein *v*_*i*_ within the PPI network for instance, its evaluation score could be calculated by PSGN score presented in Equation (10)


(10) $$\begin{array}{l} PSGN(i)=((PC(i)+NNSL(i)\times a+PeC(i)\times (1-a))\\ \times \;b+NNC(i)\times (1-b) \end{array}$$

where *a* and *b* are two weights to balance these heterogeneous features. And *a* is utilized for combining the node based and edge based biological features, *b* is set to integrate the topological features and biological features.

When integrating numerous pieces of information, several methods for identifying essential proteins require for the adjustment of parameters and the setting of an optimal one for feature combinations. In contrast, our approach proposes an adaptable parameter strategy to deal with various information based on the unique number of essential proteins that must be identified. These are the concepts: depending on the number of essential proteins we need to identify, the adaptive domain of each piece of information varies.

**Figure 2:**
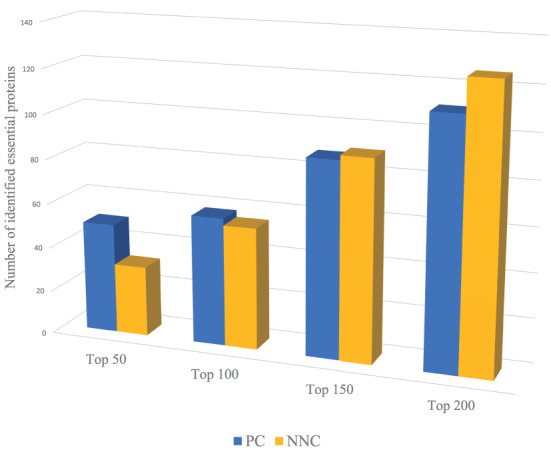
The number of essential proteins predicted by PC and NNC in different scales.

For example, we use PC and NNC two features to identify essential proteins of Yeast PPI dataset respectively. Through Figure 2, PC can capture more essential proteins compared with NNC when dealing with proteins with higher ranking positions. And for proteins with lower positions in rank, the effect of PC is not so significant as NNC. That means when we need to identify the proteins with higher ranking positions (like Top 50, Top 100), we need to assign larger weights on PC. On the contrary, to predict essential proteins with lower ranking positions (like Top 150, Top 200), NNC should be assigned with larger weights. However, most methods will give constant parameters which ignore the variation of functions of different biological information for identification when the number of essential proteins needed to be predicted changes.

In general, the effect of biological information is more reliable than topological features of network when dealing with proteins with higher ranking positions. Therefore, the weight should be adjusted adaptively according to the number of essential proteins needed to be identified. The parameter adaptive model is proposed by Equation (11)


(11) $$\begin{array}{l} P=\alpha_{i}+\beta_{i}\times input, \end{array}$$

where input is the expected number of essential proteins needed to be identified. In this research, *i* = 1 or 2, when *i* = 1, *P* = *a*, by test, we take α_1_ = 0.49, β_1_ = −0.0005, when *i* = 2, *P* = *b*, by test, we take α_2_ = 1, β_2_ = −0.0003. This parameter model means that, the weights of biological information are greater when calculating the top ranked essential proteins, especially the node-aided biological information (PC and NNSL). With the increase of input, the weight of network-based topological feature (NNC) gradually increases, and the weight of edge-aided biological information (PeC) also increases gradually.

### 2.3. Prediction of potential pan-cancer related genes

**Figure 3:**
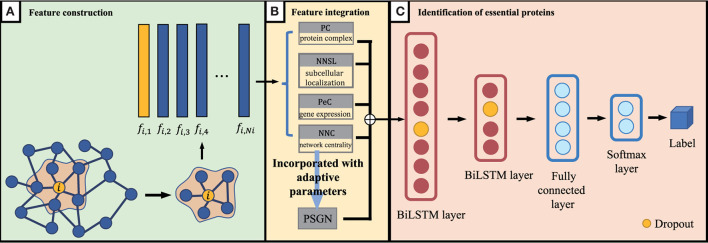
An overview of our proposed BiLSTM model. **(A)** Feature construction, **(B)** Feature integration, and **(C)** Identification of essential proteins.

As we discussed above, for proteins in the PPI network, the proteins' feature can be represented by NNC, NNSL, PC, PeC and PSGN. As it is shown in Figure 1B, the seed proteins are labeled as 1 and other proteins in yeast PPI network are labeled as 0. The final prediction results *via* enhanced BiLSTM model *via* repeated experiments as shown in Figure 3. Then the representation can be divided into training dataset and testing dataset, we sample the data from the embedding vector of pan-cancer network based on cross-validation. As shown in Figure 1, this process is trained by multiple classifiers on the sampled data. After obtaining the trained classifiers, we use them to pre- dict pan-cancer-related genes. For each predicted node, the frequency of the node can be considered as the decision metric in the training processes. Finally, the final node representation ability can be calculated by counting the frequency. We take nodes with proper frequencies as potential candidate pan-cancer genes. The whole procedures of our proposed approach AI-BiLSTM are presented in Algorithm 1.

**Algorithm 1:** BiLSTM for prediction of potential pan-cancer related genes.

**Input:** The PPI network *G* = (*V, E*), protein complex, subcellular localization, gene expression data, threshold
**Output:** The classification label for proteins;
1: Calculate PC for each protein by using Equation (1);
2: Calculate NNSL for each protein by using Equation (4);
3: Calculate PeC for each protein by using Equation (6);
4: Calculate NNC for each protein by using Equation (9);
5: Incorporate PC, NNSL, PeC and NNC by using the adaptive parameters through Equation 10 to obtain PSGN score;
6: Integrate protein feature representation enhanced by [NNC, NNSL, PC, PeC, PSGN];
7: for *i* (1 → *n*) do
8: Random select (1/n)% data as training dataset, others as test dataset;
9: Classification and fix the protein label by BiLSTM;
10: end
11: Count the frequency of the predicted essential gene (labeled 1)
12: **return** The genes with frequencies greater than the threshold.

## 3. Results and discussion

In this research, we investigate the interaction network of the model microbial Yeast, and find potential pan-cancer related genes by homologous mapping. Firstly, the LSTM model was used to categorize the essential genes in the Yeast interaction network, and then homology matching was used to further mine the disease genes. Therefore, the experimental analysis was carried out from two aspects. On the PPI datasets for yeast, we compared the performance of the novel proposed BiLSTM model with several conventional approaches. Secondly, we validated biological significance of the predicted genes through GO enrichment analysis, pathway analysis, survival analysis, clustering analysis and so forth. All of the approaches that are compared in this study adopt their default parameters. All the experiments are run on a personal computer with Windows 10 OS, Intel Core i7 2.3GHz CPU, and 16GB memory.

### 3.1. Effectiveness of the new proposed BiLSTM model

#### 3.1.1. Evaluation of PSGN

For PSGN, similar to most of validation methods for the identification of essential proteins, we also ranked all proteins by using each essential protein identification method in a descending order. And then we selected a certain number of top ranked proteins as the essential protein candidates (like top 100), after that the accuracy of identification could be computed by counting the number of true essential proteins.

**Figure 4:**
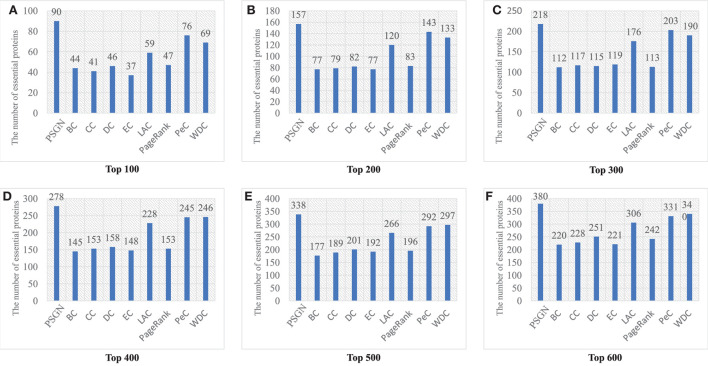
The number of essential proteins predicted by PSGN, BC, CC, DC, EC, LAC, PageRank, PeC, and WDC. **(A–F)** show the results of these methods when selecting top 100 to 600 ranked proteins as candidates of essential proteins.

Figure 4 gives a specific comparison of the results of identification of essential proteins. As shown in the figure, PSGN can identify more essential proteins compared with the other eight methods. The number of true essential proteins identified by PSGN is higher than other methods in the top 100, top 200, top 300, top 400, top 500, and top 600 proteins. In addition, by observing the results of the top 100 proteins, we find that PSGN can obtain a prediction precision of 90%, which is much higher than other methods.

**Figure 5:**
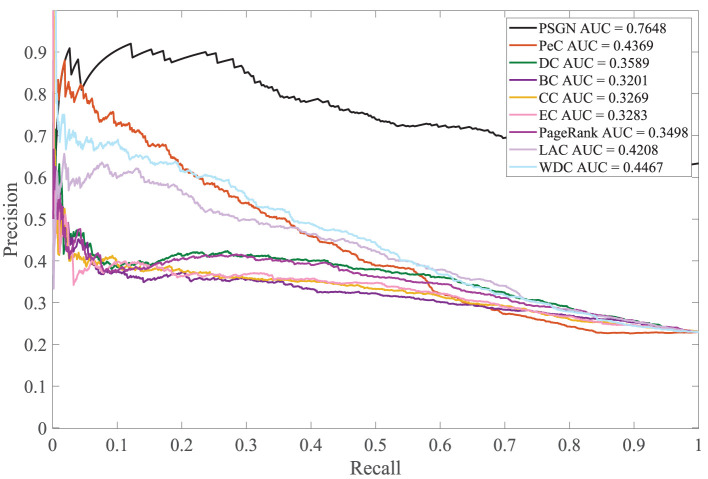
Comparison of PSGN, BC, CC, DC, EC, LAC, PageRank, PeC, and WDC using precision-recall (PR) curve method.

For better comparison, the precision-recall (PR) curve, a common methodology for evaluating the performance of essential proteins identification methods, is used in this paper. The comparison of our method with the other methods for predicting essential proteins on the Yeast PPI network by using the PR curve is shown in Figure 5. The PR curve of PSGN obtains the better result compared to the PR curves of other methods. Our method significantly exceeds other methods with the largest AUC value, illustrating the effectiveness of our method.

**Figure 6:**
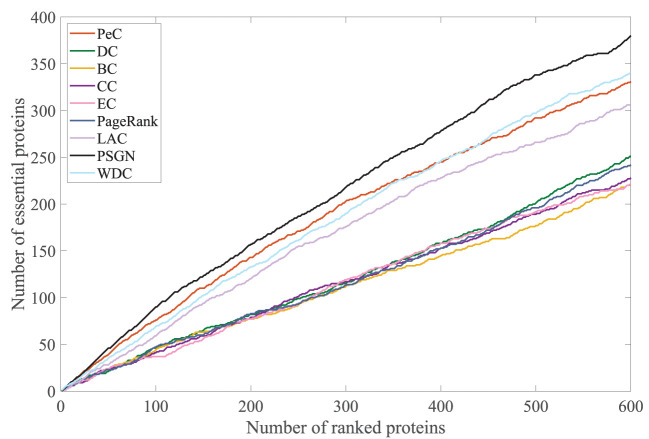
Comparison of PSGN, BC, CC, DC, EC, LAC, PageRank, PeC, and WDC using Jackknife method.

To further evaluate its effectiveness, we take the jackknife curve to compare the prediction results of our proposed method PSGN with other methods. The results are shown in Figure 6. The x-axis denotes the number of proteins ranked by each essential protein identification method and the y-axis is the number of truly identified essential proteins of each method. The areas under the jackknife curves can measure the performances of the method for identifying essential proteins. As shown in Figure 6, the jackknife curve of our proposed method PSGN can identify more essential proteins from the Yeast PPI network compared with other methods, demonstrating that PSGN is more effective and can get better results than other state-of-art methods.

**Figure 7:**
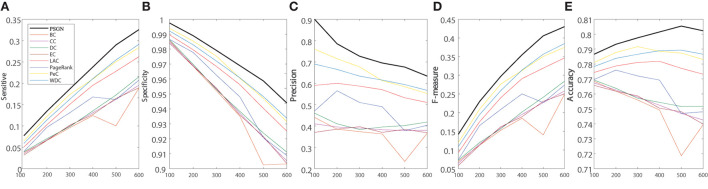
Comparative experiments on the Yeast PPI networks in terms of sensitive **(A)**, specificity **(B)**, precision **(C)**, F-measure **(D)**, and accuracy **(E)** obtained by PSGN, BC, CC, DC, EC, LAC, PageRank, PeC, and WDC.

For interpreting the advantages of our method in deeper levels, we also choose 5 widely used metrics (sensitive, specificity, precision, F-measure, and accuracy) to evaluate all the methods. Figure 7 shows the results of 5 evaluation metrics obtained by all identification methods on the PPI network of Yeast. As shown in the figure, it is obvious that our proposed PSGN can outperform other methods significantly in terms of all 5 evaluation metrics.

#### 3.1.2. Evaluate of the classified performance of BiLSTM

Machine learning algorithms like SVM, decision tree (DT), random forest (RF) and adaboost are widely used in the tasks of bioinformatics. For fair comparison with these machine learning methods, as the setting in the work of Zeng et al. (2019), we use the sequences composed of integrated biological features PC, PeC, NNSL, the topological feature NNC and the integrate feature PSGN as the input of these machine learning algorithms for training and testing. Besides, we also compared with the algorithm proposed by Zeng et al. (2019).

AI-BiLSTM proposed in this research achieved improved performance compared with other state-of-the-art algorithms with the highest value marked in bold in Table 1. Our model obtains recall, F-measure and AUC with values of 0.5674, 0.5134, and 0.6781, respectively, which are better than SVM, decision tree, random forest, Adaboost, and Zeng et al. (2019). Although our model does not show the highest values in terms of accuracy and precision and the performance is slightly weaker than SVM in these two assessments, our model owns much better recall, F-measure and AUC. In general, BiLSTM is superior to all other methods.

**Table 1:** Comparison of performance between our model and other machine learning algorithms.

**Classifier**	**Accuracy**	**Precision**	**Recall**	**F-measure**	**AUC**
SVM	**0.7654**	**0.4931**	0.3037	0.3759	0.6045
RF	0.7252	0.4295	0.5527	0.4833	0.6651
DT	0.7134	0.3809	0.3713	0.3760	0.5942
Adaboost	0.7409	0.4347	0.3797	0.4054	0.6150
Zeng et al. (2019)	0.7055	0.3802	0.4219	0.3999	0.6067
BiLSTM	0.7369	0.4803	**0.5742**	**0.5231**	**0.6829**

Besides, for verifying the significance of each feature, we make an ablation test on the features including PC, PeC, NNC, and NNSL. In the ablation experiments, we remove a feature to observe its effect on the identification of essential proteins.

Table 2 shows that NNSL takes the most crucial role in prediction of essential proteins (lowest value marked in bold). The score of accuracy, F-measure, and AUC will drop dramatically without NNSL.

**Table 2:** Experimental results for ablation test.

**Features**	**Accuracy**	**Precision**	**Recall**	**F-measure**	**AUC**
Without PC	0.7409	0.4615	**0.4918**	0.476	0.6556
Without NNSL	**0.7222**	0.4111	0.5086	**0.4547**	**0.6469**
Without PeC	0.7242	**0.3833**	0.5769	0.4606	0.6694
Without NNC	0.7311	0.4491	0.5637	0.5000	0.6736
Without PSGN	0.7340	0.4688	0.5674	0.5134	0.6781
BiLSTM	0.7369	0.4803	0.5742	0.5231	0.6829

In this section, we compared our BiLSTM with the traditional methods like DC, CC, BC, EC, NC, LAC, PeC, WDC, and PSGN. For fair comparision, 20% of top ranked proteins scored by classical methods are treated as the essential proteins, the rest are regarded as non-essential proteins. Comparing with the list of essential proteins, we can calculate the scores of accuracy, precision, recall, F-measure and AUC of each method.

As the experimental results shown in Table 3, we can find that the scores of our BiLSTM in terms of precision, recall, F-measure, and AUC are significantly higher than the results of DC, BC, CC, EC, NC, LAC, PeC, WDC, and PSGN, which also illustrates the remarkable performance of our method for identifying essential proteins.

**Table 3:** Comparison of performance between our proposed non-local GNN and other classical methods.

**Method**	**Accuracy**	**Precision**	**Recall**	**F-measure**	**AUC**
DC	0.7335	0.4050	0.3470	0.3737	0.5977
CC	0.7150	0.3580	0.3067	0.3304	0.5716
BC	0.7139	0.3550	0.3041	0.3276	0.5699
EC	0.7194	0.3690	0.3161	0.3405	0.5777
LAC	0.7563	0.4630	0.3967	0.4273	0.6299
NC	0.7469	0.4390	0.3761	0.4051	0.6166
PeC	0.7555	0.4610	0.3950	0.4254	0.6288
WDC	**0.7630**	0.4800	0.4113	0.4430	0.6394
PSGN	0.7614	0.4771	0.4301	0.4524	0.6450
LSTM-AM	0.7340	0.4688	0.5674	0.5134	0.6781
BiLSTM	0.7369	**0.4803**	**0.5742**	**0.5231**	**0.6829**

Bold values mean best scores.

### 3.2. Analyze biology significance of the new proposed method

Human disease phenotypes share corresponding orthologs in Yeast gene sets. The BiLSTM model, which was firstly established based on Yeast gene sets, has been further validated in human disease gene sets. In order to reasonably extrapolating the proposed model in microbiota-diseases, genes known to be associated formed a seed set. For the test of human disease gene prediction, we collected sets of Yeast genes whose human orthologs were linked to the same OMIM disease. Human disease phenotypes from OMIM were collapsed into major categories.

#### 3.2.1. Identification of pan-cancer related genes

In the experiments, we selected 10 kinds of cancers as the research objects, including esophageal carcinoma, pancreatic cancer, lung cancer (lung adenocarcinoma, lung squamous cell carcinoma), breast invasive carcinoma, colon adenocarcinoma, rectum adenocarcinoma, cholangiocarcinoma, gastric cancer and ovarian cancer, which can be obtained from the TCGA dataset. Due to the duplications of pathogenic genes between cancers, a total of 17,126 pathogenic genes were obtained after weight removal.

**Figure 8:**
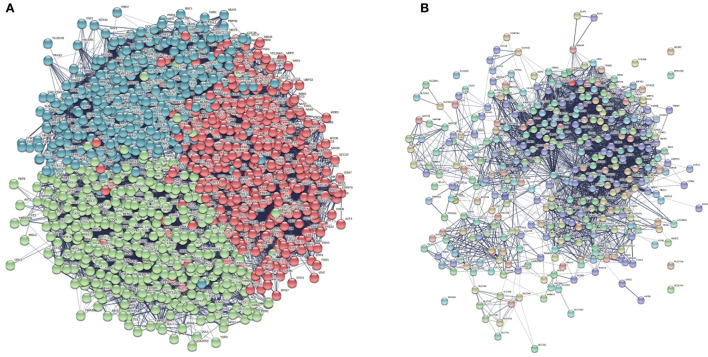
We found a total of 1,166 proteins expressed by seed genes and obtained the interaction network between these Yeast proteins screening in the background Yeast PPI network. **(A)** Generated Yeast protein interaction network. **(B)** Clustered protein interaction network with nine communities.

We believed that the common ancestor genes were similar in expression, so we did homology mapping on the background PPI network to find the homologous genes of human genes and Yeast genes. Then we take these genes as seed genes, a total of 1,166 homologous genes were found. Besides, we collected a total of 1,166 proteins expressed by seed genes and obtained the protein-protein interaction network using the STRING database. As it is shown in Figure 8A, it can be found that the corresponding Yeast proteins have a strong correlation with each other, which lays a foundation for our subsequent experiments. Through inputting the seed genes combined with the constructed PSGN features into the proposed BiLSTM algorithm, potential genes which are similar to seed genes will be predicted with corresponding scores. Predicted genes with score greater than 8 were screened out and regarded as candidate genes. By homologous mapping candidate genes, the homologous genes of these genes in human were found as the final predicted genes, and a total of 365 final predicted genes were obtained which is shown in Figure 8B. To further validate the biological significance of the predicted cancer related genes, we conducted a series of biological analysis like GO enrichment analysis, KEGG pathway analysis, clustering analysis in the following sections.

#### 3.2.2. GO enrichment analysis

For the GO items, we analyzed the relationships of final predicted genes with pan-cancers. According to the ranking of the error rate (FDP), 10 functional annotations with the largest statistical significance were obtained from Biological Process (BP), Cellular Component (CC) and Molecular Function (MF) three branches of GO datasets. As is shown in Table 4, we can find that genes are highly correlated with several important biological processes such as transcription, mRNA splicing, rRNA binding and processing, and cytokinesis, which proved the inner correlations with these predicted genes. What's more, the occurrence sites also involve several cellular sites such as nucleoplasm, ribosome and cytosols, which indicates that these predicted genes are highly related to cell development and possibility with the growth of tumors.

**Table 4:** Ten functional annotations with the largest statistical significance for three branches in GO database.

**Category**	**Term**	**Function**	***p*-value**	**FDR**
BP	GO:0006412	Translation	4.59573E-17	3.92797E-20
	GO:0000398	mRNA splicing *via* spliceosome	1.06421E-14	1.81916E-17
	GO:0061640	Cytoskeleton-dependent cytokinesis	2.2982E-11	5.89282E-14
	GO:0006749	Glutathione metabolic process	7.84992E-10	2.68373E-12
	GO:0006364	rRNA processing	1.48788E-09	6.35848E-12
	GO:0002181	Cytoplasmic translation	2.12271E-09	1.25302E-11
	GO:0034613	Cellular protein localization	2.12271E-09	1.27E-11
	GO:1903241	U2-type prespliceosome assembly	4.36481E-08	2.98449E-10
	GO:0006351	Transcription, DNA-templated	2.9991E-07	2.307E-09
	GO:0016575	Histone deacetylation	4.39654E-07	3.75773E-09
CC	GO:0005654	Nucleoplasm	1.91891E-33	5.1171E-36
	GO:0005829	Cytosol	1.29113E-15	8.91902E-18
	GO:0005940	Septin ring	1.29113E-15	1.72151E-17
	GO:0032153	Cell division site	1.29113E-15	1.72151E-17
	GO:0031105	Septin complex	1.29113E-15	1.72151E-17
	GO:0071005	U2-type precatalytic spliceosome	1.28732E-14	2.05972E-16
	GO:0005681	Spliceosomal complex	3.15875E-13	5.89633E-15
	GO:0005840	Ribosome	3.95972E-12	8.44741E-14
	GO:0046540	U4/U6 × U5 tri-snRNP complex	5.04467E-11	1.21072E-12
	GO:0005666	DNA-directed RNA polymerase III complex	4.88524E-10	1.30273E-11
MF	GO:0003735	Structural constituent of ribosome	5.78912E-16	3.91157E-18
	GO:0003899	DNA-directed 5'-3' RNA polymerase activity	7.15936E-13	7.2561E-15
	GO:0005515	Protein binding	1.02098E-11	1.3797E-13
	GO:0060090	Binding, bridging	5.14905E-09	8.69772E-11
		Proton-transporting ATP synthase activity,		
	GO:0046933	Rotational mechanism	1.20677E-06	2.44616E-08
	GO:0003743	Translation initiation factor activity	1.45514E-06	3.44121E-08
	GO:0000340	RNA 7-methylguanosine cap binding	1.51118E-06	4.08426E-08
	GO:0050291	Sphingosine N-acyltransferase activity	1.71826E-06	5.22443E-08
	GO:0015179	L-amino acid transmembrane transporter activity	2.08402E-06	7.04059E-08
	GO:0019843	rRNA binding	2.57108E-05	9.55469E-07

**Figure 9:**
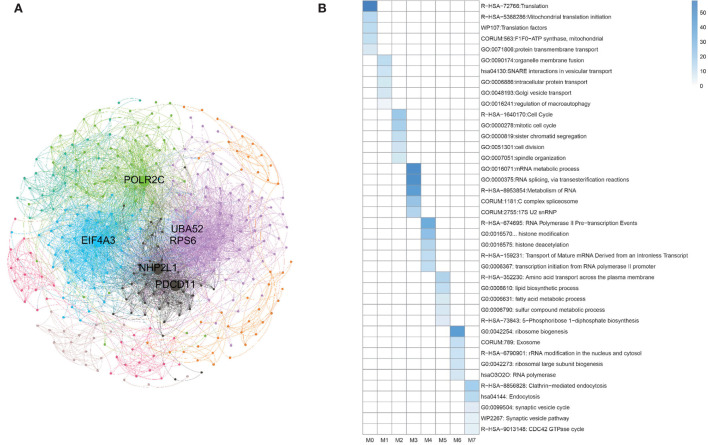
Clustering analysis for matched homologous genes in human. **(A)** Topological organization for eight modular sub-networks marked with different colors. **(B)** Enrichment biological functions of pan-cancer sub-networks. Each row represents a GO BP term and each column corresponds to a pan-cancer sub-network for each subnetwork.

Besides, during clustering analysis, eight modular subneworks *M*_0_ to *M*_7_ enriched in much more CGC genes with higher compactness structures are showed in Figure 9A. Specifically, we find that six of our predicted pan-cancer related genes are enriched in these modulars. Besides, for each module of gene lists, pathway and process enrichment analysis has been carried out with the ontology sources. The results are showed in Figure 9B.

#### 3.2.3. KEGG pathway enrichment analysis

By KEGG pathway enrichment analysis of the predicted genes, we obtained five pathways with the highest correlation with these genes like Proteasome (map03050), Valine, leucine and isoleucine degradation (map00280), Terpenoid backbone biosynthesis (map00900), Mismatch repair (map03430), and Glutathione metabolism (map00480). Among these pathways, the proteasome pathway was the most enriched pathway, which are usually used as an inhibitor in the cancer therapy.

#### 3.2.4. Survival analysis

**Figure 10:**
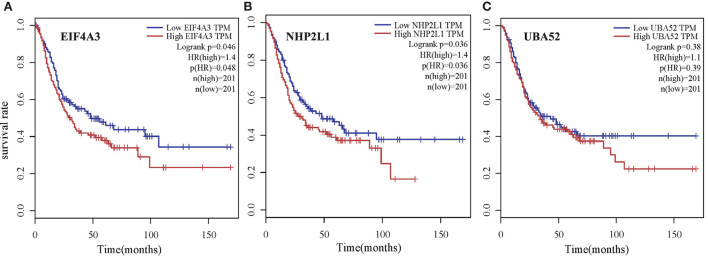
Survival analysis of three genes with the highest moderate prediction of human genes, which are closely related to RNA metabolic function for Bladder urothelial carcinoma (BLCA) patients. **(A)** EIF4A3, **(B)** NHP2L1, and **(C)** UBA52.

To verify the biological significance of the experimental results, we conducted further survival analysis. As shown in Figure 10, EIF4A3, NHP2L1, and UBA52 are the three genes with the highest moderate prediction of human genes, which are closely related to RNA metabolic function. Here, we carried out a survival analysis of these three genes, respectively, and it can be seen from the results that all these three genes have a significant impact on the survival time of Bladder urothelial carcinoma (BLCA) patients, which verifies the performance of the new proposed prediction method from the respective of homologous matching.

## 4. Conclusion

High-throughput techniques and machine learning approaches, combined with an increasing understanding of the microbiota and their collective genome from preclinical and large-scale clinical studies, offer exciting opportunities for modernizing microbe-based strategies from untargeted to precision microbiome-centered therapies. Essential proteins have drawn attention for their crucial roles in controlling signal transduction, individual variation in treatment response, and a wide range of other microbiome-related processes. The properties and purposes of biological data used to identify critical proteins are explored in this study. In light of the findings, we suggest a linear adaptive model PSGN, which may adaptively modify the weights for balancing each type of biological or topological property. We have demonstrated that the NNSL feature is significantly more important than other features through experimental validation. Moreover, the new algorithm PSGN improved the ability to represent features discriminatively. In the experiments, we first contrasted the PSGN with established methods including PageRank, DC, BC, CC, EC NC, LAC, PeC, and WDC. The results demonstrated that PSGN outperforms the other approaches in terms of overall performance. Furthermore, we evaluate our BiLSTM with machine learning methods and the most recent deep learning-based methods. The results of experiments may potentially establish the capability of the new proposed BiLSTM. Our suggested models for biological information have considerable generality, making them suitable for integrating almost all biological features. In the future, we will continue to test and search for more suitable biological information for identifying essential proteins in more species.

## Data availability statement

The original contributions presented in the study are included in the article/supplementary material, further inquiries can be directed to the corresponding author.

## Author contributions

CW: conceptualization. HZ and HM: methodology. HZ and KC: software. CW, TG, YW, YY, and ZL: validation. HZ and YZ: writing—original draft preparation. All authors have read and agreed to the published version of the manuscript.

## Funding

This work was partially supported by the National Natural Science Foundation of China (12126367 and 12126305), Chen Xiao-Ping Foundation for the Development of Science and Technology of Hubei Province (CXPJJH12000002-2020058), the Hubei Provincial Natural Science Foundation of China (2015CFA010), Fundamental Research Funds for the Central Universities, China University of Geosciences (Wuhan) (CUGGC02), and Shanghai Municipal Science and Technology Major Project (2018SHZDZX01), Key Laboratory of Computational Neuroscience and Brain-Inspired Intelligence (LCNBI), and ZJLab.

## Conflict of interest

The authors declare that the research was conducted in the absence of any commercial or financial relationships that could be construed as a potential conflict of interest.

## Publisher's note

All claims expressed in this article are solely those of the authors and do not necessarily represent those of their affiliated organizations, or those of the publisher, the editors and the reviewers. Any product that may be evaluated in this article, or claim that may be made by its manufacturer, is not guaranteed or endorsed by the publisher.
